# A Novel Robust Method Mimicking Human Substratum To Dissect the Heterogeneity of Candida auris Biofilm Formation

**DOI:** 10.1128/spectrum.00892-23

**Published:** 2023-07-13

**Authors:** Biswambhar Biswas, Aishwarya Rana, Nidhi Gupta, Ishaan Gupta, Rekha Puria, Anil Thakur

**Affiliations:** a Laboratory of Protein Translation and Fungal Pathogenesis, Regional Centre for Biotechnology, Faridabad, Haryana, India; b School of Biotechnology, Gautam Buddha University, Greater Noida, Uttar Pradesh, India; c Department of Biochemical Engineering and Biotechnology, Indian Institute of Technology Delhi, Delhi, India; University at Buffalo, State University of New York

**Keywords:** *Candida albicans*, *Candida auris*, *Candida glabrata*, EPS, gelatin, biofilms

## Abstract

Candida auris is a pathogen of urgent threat level as marked by the CDC. The formation of biofilms is an essential property of this fungus to establish infection and escape drug treatment. However, our understanding of pathogenesis through biofilm is hampered by heterogeneity in C. auris biofilms observed in different studies. It is imperative to replicate *in vivo* conditions for studying C. auris biofilm formation *in vitro.* Different methods are standardized, but the surface used to form biofilms lacks consistency as well as the architecture of a typical biofilm. Here, we report an *in vitro* technique to grow C. auris biofilms on gelatin-coated coverslips. Interestingly, C. auris cells grown on gelatin-coated coverslips either on modified synthetic sweat media or RPMI 1640 resulted in similar multilayer biofilm formation with extracellular polymeric substances (EPS). This method is also consistent with the biofilm formation of other *Candida* species, such as Candida glabrata and Candida albicans. Biofilms of C. glabrata developed through this method show pseudohyphae and EPS. This method can be used to understand the molecular basis of biofilm formation, associated pathogenesis, and drug tolerance. The technique is cost-effective and would thus serve in rightful screening and repurposing drug libraries for designing new therapeutics against the less-studied high-alarm pathogen C. auris.

**IMPORTANCE** Heterogeneity is seen when multidrug-resistant C. auris biofilm is cultured using different reported methods. Biofilm formed on the gelatin surface mimics the condition of a host environment that has multilayers and EPS. This method has feasibility for drug screening and analyzing biofilms through three-dimensional (3D) reconstruction. This *in vitro* biofilm formation technique is also exploited to study the formation of biofilm of other *Candida* species. The biofilms of C. glabrata and C. albicans can also be correctly mimicked using gelatin in the biofilm-forming environment. Thus, the novel *in vitro* method for biofilm formation reported here can be widely used to understand the mechanism of biofilm formation, related virulence properties, and drug tolerance of C. auris and other *Candida* species. This simple and low-cost technique is highly suitable for screening novel inhibitors and repurposed libraries and to design new therapeutics against *Candida* species.

## INTRODUCTION

Candidiasis is a major concern among individuals having weak immune systems and comorbidities. Hospitalized patients after organ transplantation or bone marrow transplantation and virus-infected patients undergo immune-suppressive drug therapy to tackle immune hyperactivation. This and the required prolonged hospital stay make them prone to pathogen infection/hospital-acquired infection. In 2009, a fungus of the *Candida* family was isolated from the ear canal of a 70-year-old Japanese woman and was named Candida auris (1). It has emerged worldwide in different countries and reports of drug resistances have come from 32 countries (2–4). First, it emerged parallelly across 3 continents and soon after in North America, probably during travel (5). A fifth clade has also emerged and is from Iran (6). The identification of this fungus was crucial, as it mimics the morphology of other fungi. It was accurately distinguished from other fungi using mass spectrometers (7). It can cause ear infection, systemic infection or fungemia, wound infection, lung infection, urinary tract infection, and gut infection (8–11). WHO has declared the fungus on the topmost priority list. The fungus can resist the treatment of various antifungals and be pan-drug resistant. The biofilm form of the pathogenic organism is highly drug resistant. As per CDC guidelines, the biofilm of Candida auris is resilient. Biofilms of C. auris are resistant to common ammonium salt-based disinfectants and can withstand other harsh conditions (12). Thus, the study of biofilm in an infection scenario is essential.

The prevalence of drug resistance against existing drugs demands a platform for the robust screening of novel inhibitors. Additionally, potential inhibitors identified through drug repurposing need to be validated through various *in vitro* and *in vivo* screening methods. However, to perform such screening, it is imperative to establish a robust and reproducible method to form a biofilm model of the infection that faithfully replicates *in vivo* conditions. This is one of the major challenges currently faced by researchers, as C. auris biofilm formation manifests different phenotypes when cultured in different conditions (13, 14). This is primarily driven by a lack of understanding of the principles behind C. auris biofilm formation. The environmental conditions in which *in vitro* biofilm formation occurs can have a significant impact on the results and need to be carefully controlled to obtain accuracy. Currently, the available methods of biofilm formation do not use a standardized surface that would ensure reproducibility in biofilm proliferation. For example, Sherry et al. (14) and Horton et al. (15) describe the architecture of C. auris biofilm grown *in vitro* on a Thermanox (polystyrene) coverslip consisting of a single layer of yeast cells, which is not the typical biofilm architecture (14, 15). While Horton et al. further described the use of porcine skin surface and synthetic sweat media to proliferate multilayered biofilm (15, 16), these available *in vitro* methods lack cost-effectiveness, especially in the case of the large-scale screening of libraries.

Given that C. auris primarily colonizes the skin, we offer a novel *in vitro* method for growing C. auris biofilms on gelatin-coated coverslips (derived from bovine skin). We reasoned that since gelatin is a derivative of collagen and is made from skin, tendon, and bone extracts, it can mimic the matrix of mammalian cells and thereby help to replicate the natural scenario (17, 18). We demonstrate the application of this method on C. auris to form a heterogeneous multilayer biofilm. Further, the architecture of the fungal biofilm was validated under a scanning electron microscope (SEM). Further, we assessed the feasibility of gelatin-coated coverslips for drug screening, and these can be quantitatively measured using viability assays and thickness measurement using the confocal scanning laser microscopy technique. It is interesting to note that this *in vitro* biofilm formation technique can also be exploited to study the biofilm formation of Candida glabrata and Candida albicans.

## RESULTS

### C. auris forms multilayered high-burden biofilm on gelatin.

**FIG 1:**
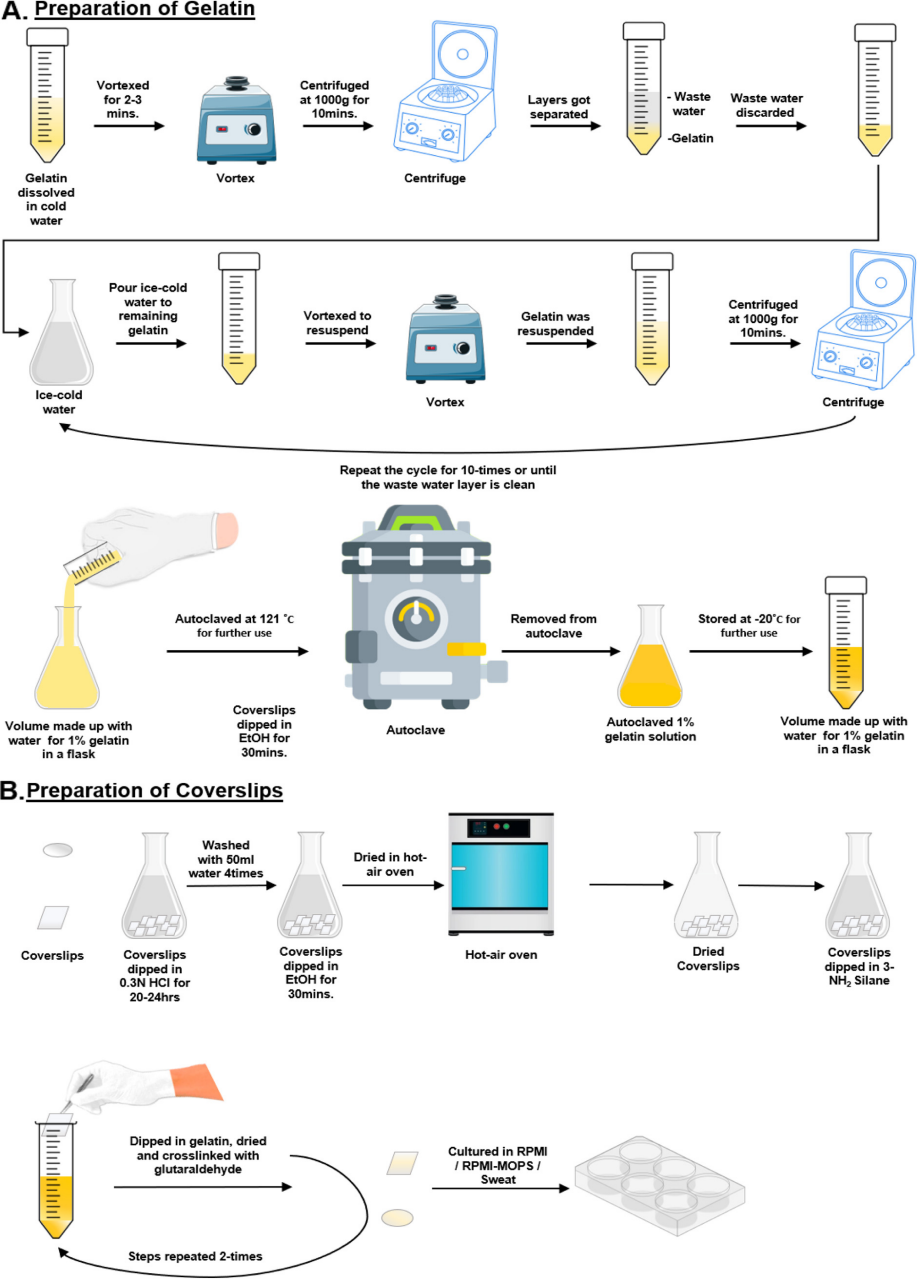
Flow chart for gelatin-based culturing of C. auris. (A) Diagrammatic view of gelatin preparation by washing the gelatin several times and then autoclaving it for coating glass coverslips. (B) Diagrammatic view of coating of gelatin on the surface of coverslips for biofilm formation.

**FIG 2:**
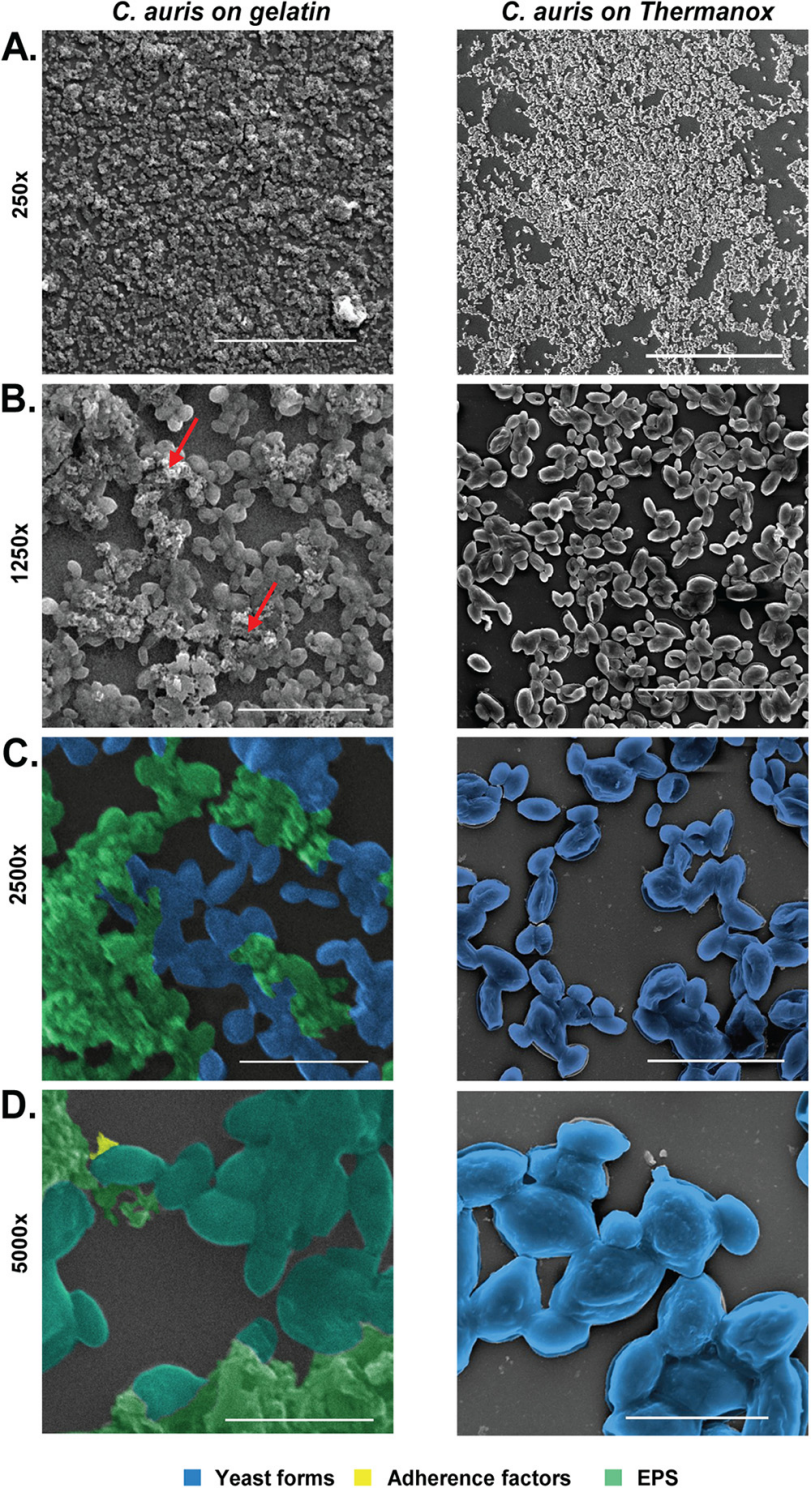
Comparison of SEM images of biofilm of C. auris on the gelatin-coated surface and Thermanox coverslip. (A) SEM imaging was done at 250× (scalebar 1 in. = 300 μm) on gelatin-coated coverslips. (B) 1,250× (scalebar 1 in. = 50 μm) on gelatin-coated coverslip with dense biofilm and EPS represented by the canalicular system of compact trabeculae with spiny surface marked with red arrows and monolayer in Thermanox coverslips. (C) 2,500× and pseudocoloring showing different cell morphologies like yeast forms, EPS (scalebar 1 in. = 30 μm). (D) Further magnification to 5,000× reveals attachment junctions marked as adherence factors with yellow color seen in biofilms grown in gelatin-coated coverslips. Also, C. auris yeast cells in the biofilms are found to be covered with EPS (Scalebar 1 in. = 10 μm).

Biofilm plays a crucial role in drug tolerance and pathogenicity of C. auris. Based on the proposition that C. auris colonizes skin efficiently and gelatin mimics skin architecture, we have developed an *in vitro* method to culture C. auris on a gelatin-coated coverslip. Collagen is the most abundant protein inside the body and acts as a good substrate for adherence and provides the basal layer for different types of tissue formation. Gelatin is a hydrolyzed form of collagen that also provides mesh to adhere and also may help as an available source of nutrition. Gelatin is an economical alternative to Thermanox coverslips, as it mimics the *in vivo* system of the host closely. We have assessed the prevailing techniques to understand the biofilm-forming capability of gelatin (16). We have also incorporated a technique to measure biofilm thickness using microscopy that could reduce handling errors. We started by coating the coverslips with gelatin. Dried gelatin on the coverslip surface was fixed with glutaraldehyde. Another coating was made to ensure coverage of any uncoated surface as well as to make the coating even and equal (Fig. 1A and B). Dried coverslips were used to culture the biofilm. The biofilm was cultured in a 6-well plate on gelatin-coated coverslips in RPMI 1640. To compare the potential of biofilm formation on gelatin and Thermanox coverslips, we grew the C. auris biofilm on both surfaces using RPMI 1640 medium. To evaluate the architecture and cellular morphology of biofilm, we imaged them using scanning electron microscopy. We have noticed a higher density of biofilm formation on gelatin-coated coverslips than on Thermanox coverslip surfaces (Fig. 2A and B). Biofilm was grown on a gelatin coverslip surface comprised of a multilayer dense network of yeast cells embedded in an extracellular polymeric substance (EPS). In contrast, C. auris biofilm grown on a Thermanox coverslip consists of a single layer of yeast cells, and a lower burden of biofilm was observed as per previous reports (19). To show morphologies of yeast cells and EPS, these were represented in the SEM images using different pseudocolorings (Fig. 2C and D).

### Gelatin-coated surface is a suitable platform for drug screening against C. auris biofilm.

**FIG 3:**
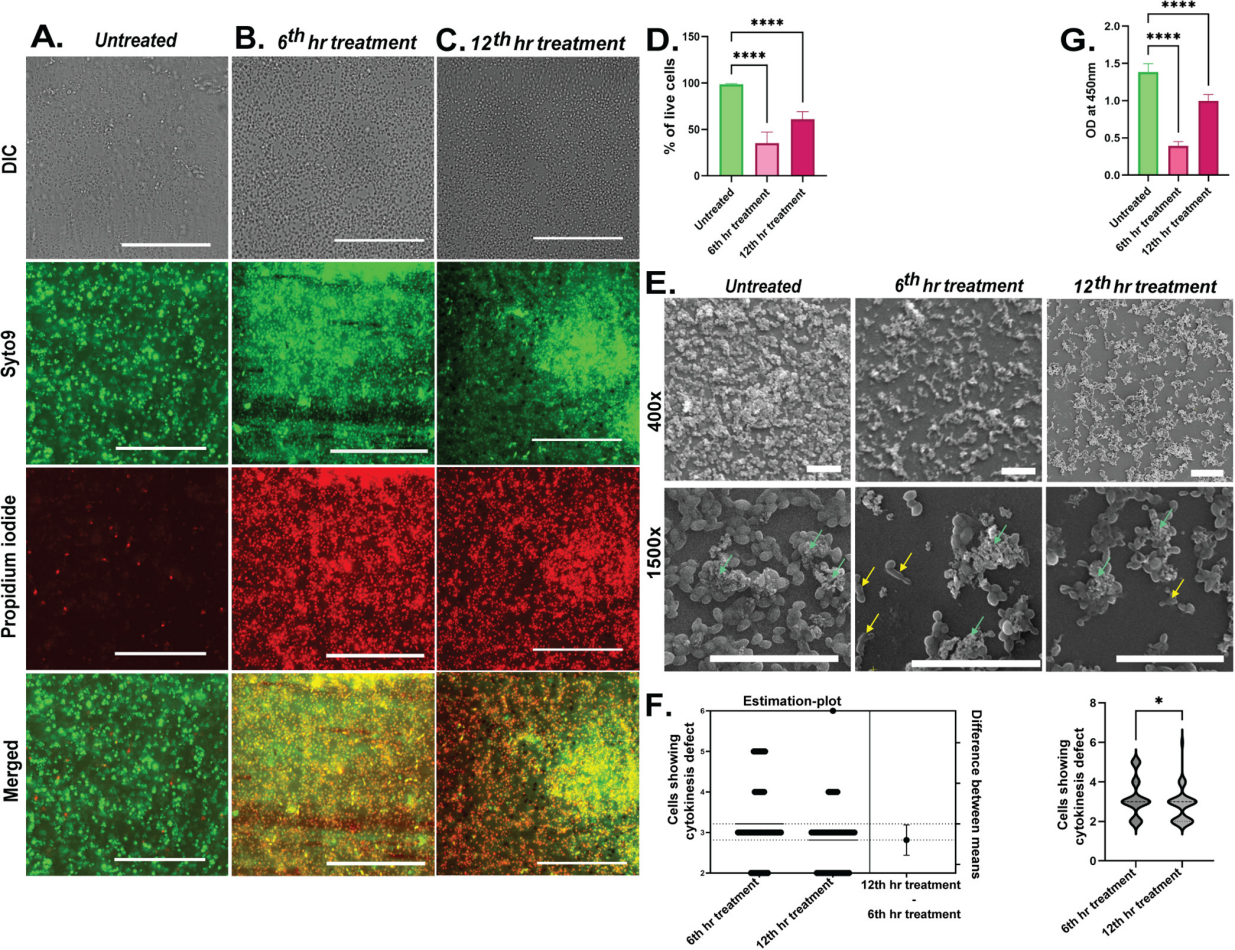
Live and dead cell staining, thickness measurement, metabolic activity, and morphological analysis of C. auris biofilms cultured on the gelatin-coated surface of coverslips in various conditions. (A to C) Representative images of live and dead cell staining of C. auris biofilm from 3 biological and 3 technical replicates as follows: untreated condition (A), fluconazole treatment after 6th hour of biofilm formation (B), and fluconazole treatment after 12th hour of biofilm formation (C). The scalebar is fixed to 100 μm. (D) Percentage of live cells from 3 biological and 3 technical replicates of untreated and treatment groups were plotted (±standard deviation [SD]; ****, *P* < 0.0001%). (E) Cells imaged using a scanning electron microscope show dense biofilm formation with EPS and canalicular architecture in the untreated group. In the treatment groups, it is seen that cells are reduced in number and show cytokinesis defect. Yellow arrows indicate cytokinesis defects, and green arrows indicate EPS. (F) Comparison of the 14 μg/mL fluconazole treatment and untreated groups; the 6th hour treatment group shows more cytokinesis defect than the 12th hour treatment group as observed in 6 different fields from 3 biological replicates (±SD; *, *P* < 0.0280%). (G) The metabolic activity measured through XTT assay of C. auris biofilm formed over gelatin-coated coverslip in untreated condition, fluconazole treatment after 6th hour of biofilm formation and fluconazole treatment after 12th hour of biofilm formation. Three biological replicates, each with 2 technical replicates, were analyzed (±SD; ****, *P* < 0.0001%).

Here, to further assess the feasibility of the gelatin-coated coverslips for drug screening, we have used known antifungal fluconazole against C. auris. The log-phase cells were inoculated into RPMI 1640, and cells were adjusted to an optical density at 600 nm (OD_600_) of ~0.5. These cells were incubated at 37°C for 2.5 h in order to adhere to the gelatin-coated coverslip. After 2.5 h, the medium was discarded from the wells and washed with phosphate-buffered saline (PBS) once. This marked the 0 h or time point zero of biofilm formation. The wells were again replenished with fresh RPMI 1640 medium and left for 48 h for biofilm formation. There were 3 groups as follows: the 6th hour treatment group, 12th hour treatment group, and untreated. In the 6th hour treatment group, the 14 μg/mL fluconazole was added after the adhered cells were incubated for 6 h. Similarly, in the 12th hour treatment group, treatment was given after the adhered cells were incubated for 12 h. These treatment groups were further left for incubation for the remaining time of biofilm formation. The untreated group was not given any treatment, and the adhered cells were incubated for 48 h. The treatment times were based on an earlier report that the early stage of biofilm formation ends at the 4th hour, which is succeeded by the intermediate stage which ends at the 12th hour, after which the formation of mature biofilm occurs (20). So, we gave fluconazole treatments at the beginning of the intermediate phase of biofilm formation and at the beginning of the maturation stage. After 48 h of biofilm formation, all three groups were assessed for cell viability through LIVE/DEAD *Funga*Light yeast viability kit assay. The stained slides were imaged in a fluorescence microscope. Here, we assessed the fluorescence area covered by green puncta from Syto 9 for cell analysis and the red fluorescence area from propidium iodide (PI) for dead cell analysis. The captured images were split into two separate channels in FIJI. The image of each channel is thresholded to define the background and the puncta. After thresholding, the area covered by the puncta was shown in white and the background in dark. The area taken up by each white particle was measured, and results were saved for each of the channels. These analyses were done for untreated and treated groups to calculate the live and dead cells of C. auris biofilm. Representative images of Syto 9 and PI staining of C. auris biofilm from 3 biological and 3 technical replicates in untreated conditions, fluconazole treatment after the 6th hour of biofilm formation, and fluconazole treatment after the 12th hour of biofilm formation were shown in Fig. 3A to C, respectively. The percentage of live cells was plotted in the graph (Fig. 3D). In the untreated sample, the percentage of live cells was ~98%, which decreased to 35.16% in the 6th hour treatment group. The 12th hour treatment group was found to have ~61% of live cells. This implies that most of the cells were dead when the treatment was given in the 6th hour, which is the early intermediate stage of biofilm formation. More live cells and fewer dead cells were found when treatment was given in the 12th hour, which is the beginning of the maturation phase of biofilm. Next, we utilized scanning electron microscopy to assess the architecture of these treated C. auris biofilms. There were a greater number of cells in the untreated group than in the 6th hour treatment and 12th hour treatment group (Fig. 3E). The prominent cytokinetic defects among the 6th hour treatment group cells were observed, wherein ~5 cells failed to detach and appeared in chains. This defect was reduced in the 12th hour treatment group (Fig. 3F). Similar biofilm inhibition observations were made with the Tor inhibitors in our lab (21). Further validation of results was achieved through cell viability assay using 2,3-bis-(2-methoxy-4-nitro-5-sulfophenyl)-2H-tetrazolium-5-carboxanilide salt (XTT). A similar scenario was replicated where the 6th hour treatment group had a less viable biofilm and lower absorbance of ~0.39 compared to the 12th hour absorbance of ~0.99 and untreated group absorbance of ~1.38 when OD was measured at 450 nm (Fig. 3G).

**FIG 4:**
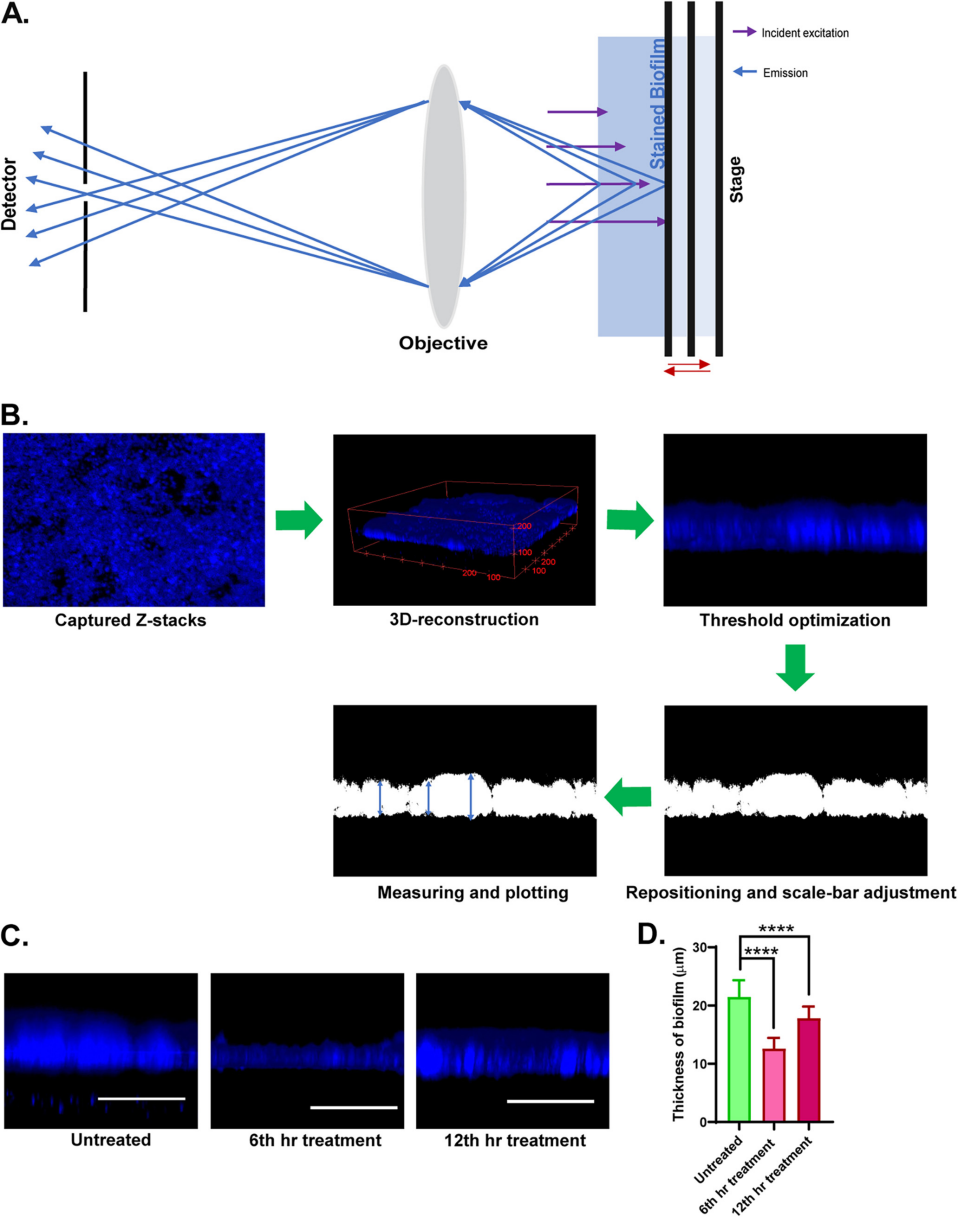
Study plan to measure the thickness of biofilm and comparison of C. auris biofilm cultured on gelatin-coated coverslips. (A) Schematic representation to get the information on biofilm thickness. (B) Flowchart for 3D reconstruction and measuring the biofilm thickness. Thickness measurement of biofilm formed on a gelatin-coated coverslip in panel C. (C) Untreated and fluconazole treatment after 6th hour and 12th hour of biofilm formation. (D) Comparison of biofilm thickness of the 6th hour, 12th hour treatment, and untreated groups. For each group, values were plotted using 3 biological replicates each having 2 fields from technical replicates and measurements from 5 random regions from each (±SD; ****, *P* < 0.0001%).

To unveil the thickness of biofilm, we analyzed the three-dimensional (3D) reconstruction of the biofilm. The two-dimensional (2D) image obtained by using a confocal laser scanning microscope contains an observation of one field view with a particular depth. The light coming from different planes has different focal points, and hence, it cannot be captured while keeping the stage fixed. So, we stacked images captured in different planes of a field by moving the stage in the *z* axis and analyzed the thickness (Fig. 4A and B). We analyzed the thickness of biofilm formation by giving the same treatment of 14 μg/mL fluconazole in the 6th hour and 12th hour of biofilm formation and incubated all groups for the remaining time of biofilm formation. We observed that the untreated group has a thicker biofilm compared to the 6th and 12th hour treatment groups by staining with calcofluor white (CFW) as seen in Fig. 4C. The data gathered from multiple biological replicates was compiled in a graph for comparison. The average thickness of the untreated group was 21.4 μm, whereas it was 12.59 μm and 17.83 μm for the 6th and 12th hour treatment group, respectively (Fig. 4D). The results correlated with the inhibitory effect of fluconazole on biofilm formation. Taken together, these results suggest that biofilm formed by C. auris on the gelatin surface is suitable for drug screening against C. auris biofilm *in vitro*.

### C. auris biofilm exhibits similar proliferation in RPMI and modified sweat medium on the gelatin-coated surface.

**FIG 5:**
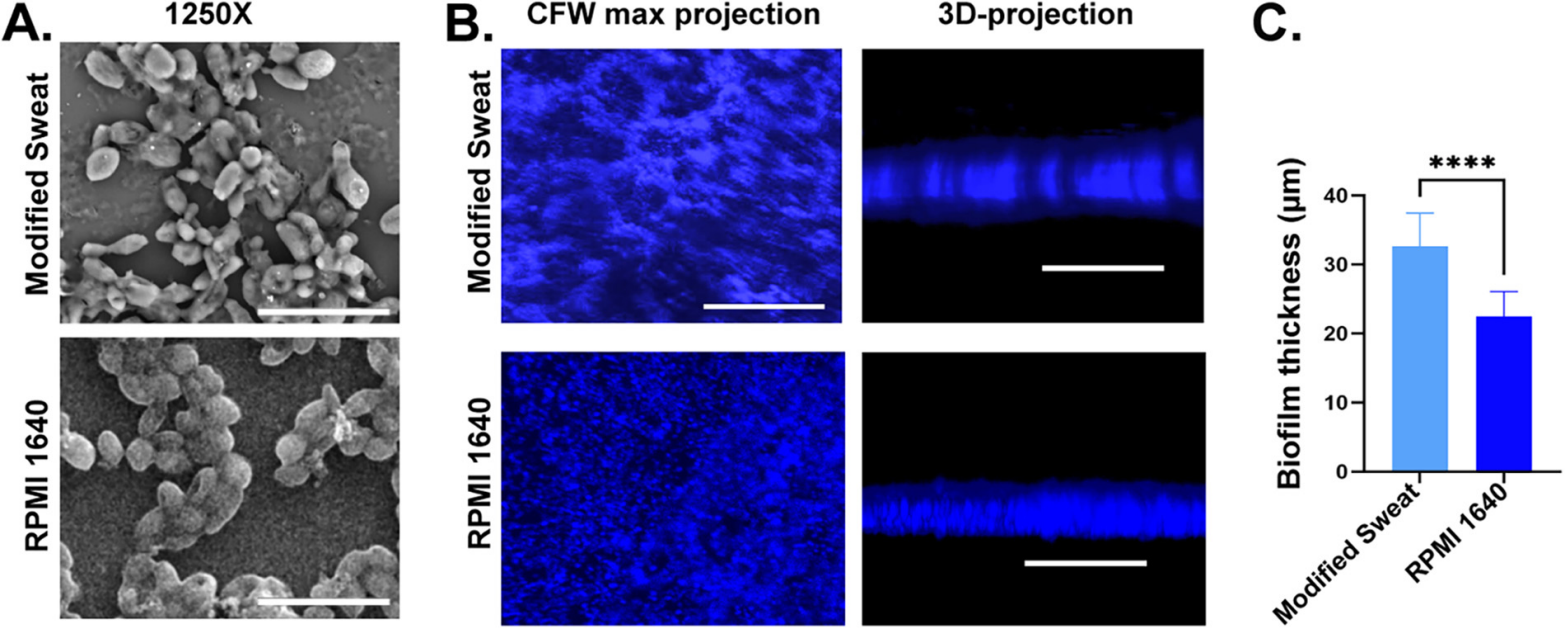
Biofilm architecture in artificial sweat medium and RPMI cultured on gelatin-coated coverslips. (A) SEM imaging of cells grown on RPMI medium and sweat medium. (B) Biofilm formation of C. auris cells cultured in sweat media and RPMI 1640 and 3D projection; scalebar is 100 μm. (C) Comparison of biofilm thicknesses between cells cultured in modified sweat medium and RPMI 1640 medium. For each group, values were plotted using 3 biological replicates each having 2 fields from technical replicates and measurements from 5 random regions from each (±SD; ****, *P* < 0.0001%).

To understand the mechanism of skin colonization, we analyzed the formation of biofilm on the sweat medium. The sweat medium mimics the *in vivo* condition of the skin. The biofilm formation on skin and skin wound infection can be replicated (17, 22). The biofilm grown on the gelatin-coated coverslip in modified sweat medium at 37°C inside a CO_2_ incubator was used to mimic the *in vivo* condition of the skin. RPMI 1640 medium was used to form biofilm to mimic other *in vivo* conditions (23). This is important, particularly in the case of C. auris, where it shows different growth phenotypes in different conditions (13, 15, 16). To differentiate the biofilm formation in two different conditions, we grew C. auris biofilm in both modified synthetic sweat medium and RPMI 1640 on a gelatin-coated coverslip and monitored them by scanning electron microscopy. For modified sweat medium formation, we obtained total lipids from fetal bovine serum (FBS) using the MeOH/chloroform extraction method (Table 1). Modified sweat medium-grown biofilm proliferated as a multilayer and thickened biofilm composed entirely of yeast cells. Intriguingly, the biofilms were architecturally similar in both modified sweat and RPMI 1640 media as seen in SEM images (Fig. 5A), but the thickness of the biofilm differed as analyzed three-dimensionally (Fig. 5B). The mean biofilm thickness was ~22 μm and ~32 μm in RPMI 1640 medium and modified sweat medium, respectively (Fig. 5C). These findings suggest C. auris exhibits an enhanced tendency to proliferate on gelatin even under different growth conditions.

**TABLE 1:** Modified sweat medium

Component	Concentration (mg/mL)
Sodium chloride	1.75
Calcium chloride	0.6
Lactic acid (88%)	1.441
Urea	1.201
Hydrolyzed fatty acids	0.02
Cholesterol	0.0006
Glycine	0.00125
l-leucine	0.00026
l-cysteine	0.00003
l-serine	0.00303
l-alanine	0.00081
l-arginine	0.00001
l-histidine	0.00043
l-threonine	0.00066
l-valine	0.0032
l-isoleucine	0.00020
l-lysine	0.00021
l-phenylalanine	0.00021
l-tyrosine	0.00034
l-asparagine	0.00022
l-glutamic acid	0.00023
l-methionine	0.00007
l-glutamine	0.00003
l-aspartic acid	0.00055
l-ornithine	0.00086
l-citrulline	0.00073
l-ascorbic acid	0.00001
d-glucose	1.08
Sodium pyruvate	0.55
Sodium bicarbonate	0.925
NaH_2_PO_4_	0.0022
K_2_HPO_4_	1.1312
Magnesium sulfate	0.012

### Candida species form high-burden biofilm on gelatin-coated surfaces.

**FIG 6:**
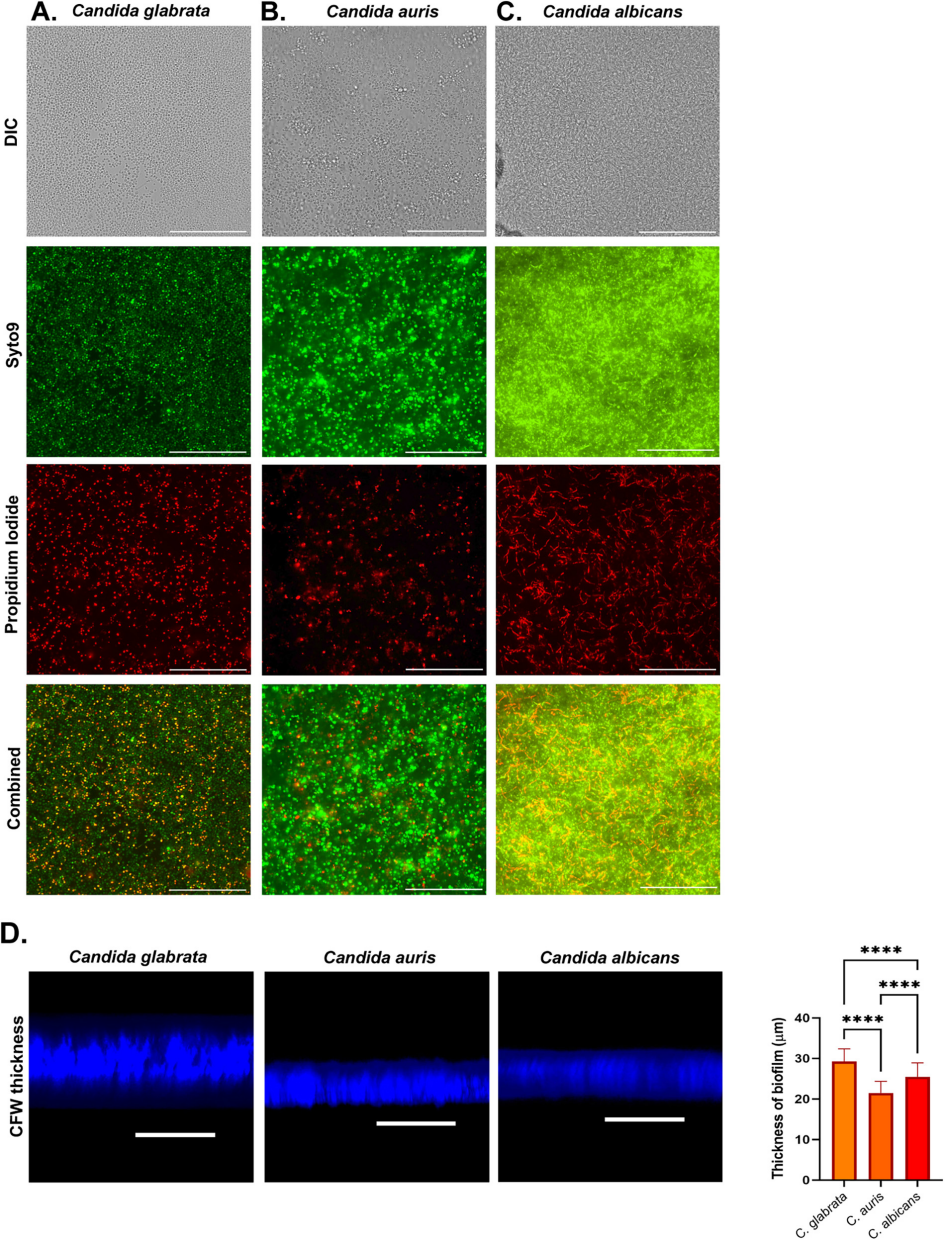
Comparison of biofilm of C. glabrata, C. auris, and C. albicans cultured on gelatin-coated-coverslip. Biofilm formed on gelatin-coated coverslips and using live and dead staining and thickness measurement to validate the techniques on C. glabrata (A), C. auris (B), and C. albicans (C). Scalebar is 100 μm. (D) Comparison of biofilm thickness of C. glabrata, C. auris, and C. albicans (±SD; ****, *P* < 0.0001%).

**FIG 7:**
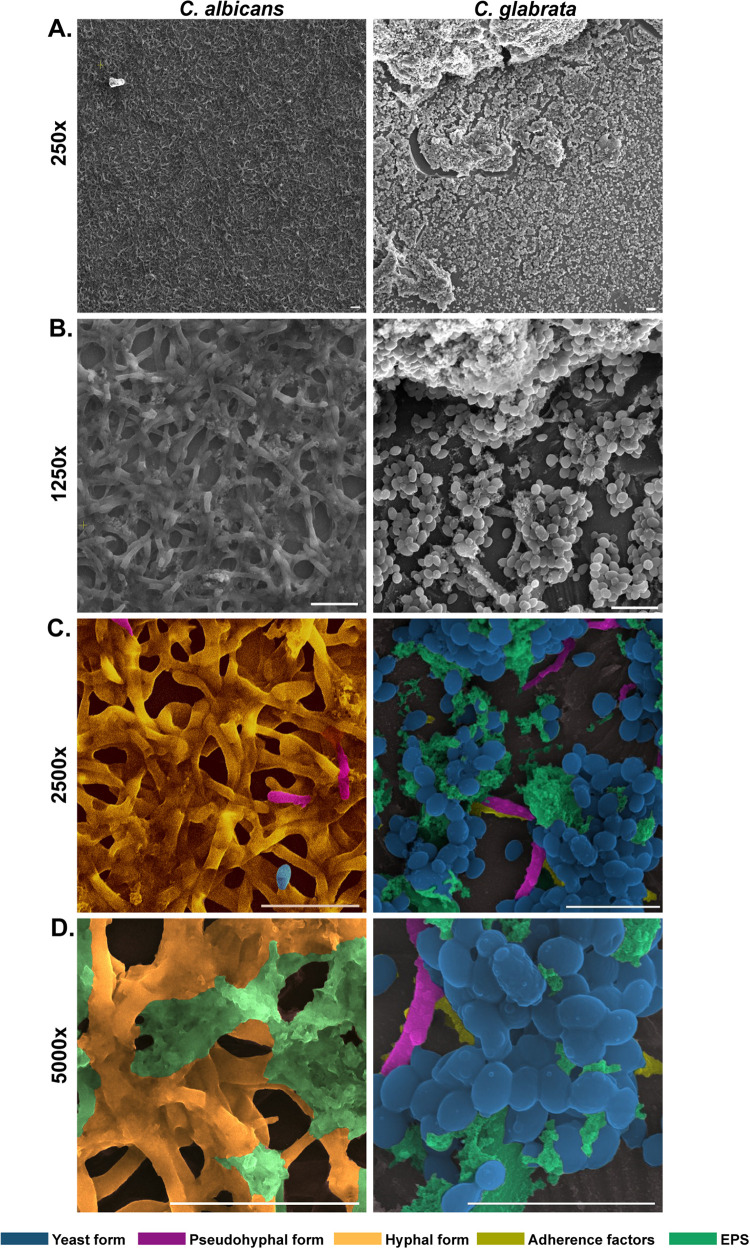
SEM imaging of biofilm formation of C. albicans and C. glabrata on gelatin-coated surface imaged at 250× (A), 1,250× (B), 2,500× (C), and 5,000× (D) magnification. Pseudocoloring shows different cell morphologies and color coding of different morphologies are represented in the figure.

We have also checked the ability of the biofilm formation by other *Candida* species, such as C. glabrata and C. albicans on gelatin-coated coverslips. For C. albicans, we used RPMI-morpholinepropanesulfonic acid (MOPS) at pH 7.0, and for C. glabrata, RPMI 1640 was used. We used LIVE/DEAD *Funga*Light yeast viability kit staining to identify live and dead cells of C. glabrata, C. auris, and C. albicans (Fig. 6A to C). Using CFW staining and z-stacking, we have shown that C. glabrata, C. auris, and C. albicans have mean biofilm thicknesses of ~29.3 μm, ~21.5 μm, and ~25.5 μm, respectively (Fig. 6D). Further, we analyzed the architecture of the biofilm of C. albicans and C. glabrata by scanning electron microscope (Fig. 7A and B). While the biofilm of C. albicans consists of dense hyphae and yeast forms, C. glabrata showed a dense layer of yeast cells and a few pseudohyphal forms, congruent with their known behavior (24, 25). These morphologies are represented using different pseudocolorings in Fig. 7C and D. Thereby, the *in vitro* method reported here can also be utilized in biofilm formation studies of other *Candida* species.

## DISCUSSION

Candida auris is an organism that shows different behavior when cultured in different conditions. So, the more that we can mimic the *in vivo* scenario, the better the visualization of the human infection scenario. The technique of using gelatin for biofilm culturing is a cost-effective alternative to using commercial polypropylene coating. Other materials like elastomer, epoxy resin, polyurethane, polystyrene, and artificial leather lorica can also mimic skin due to their friction-related properties but under specific conditions, and most of these models have a major limitation in that they do not interact with water (17, 18). Nonetheless, gelatin mimics the natural substratum for cell adherence (17). This can help boost drug discovery for labs, where acquiring biosafety permissions for using the skin requires special provisions, etc. We can use prevalent microscopical techniques like live and dead cell quantification, SEM, etc., with gelatin coating to study biofilms. The microscopic techniques used here to determine biofilm thickness and comparison are user-friendly. Also, studies can be performed by less experienced hands, as this does not involve multiple washing, staining, and destaining steps. Thereby, reducing the chance of errors and variation between replicates. Gelatin coating can also be done on 96-well or other plates to mimic the natural host environment. Initially, we anticipated the use of gelatin as a close mimic of skin constituents/architecture, an environment preferred by C. auris for colonization and biofilm formation. The dense proliferation of biofilms in modified sweat medium with gelatin-coated surfaces authenticated our hypothesis. The success of the development of biofilms of other *Candida* species by this *in vitro* technique highlights the fact that gelatin offers a very accurate natural substratum that supports the biofilm formation for other *Candida* species. Moreover, Candida glabrata also does not show the true biofilm architecture (multilayer and pseudohyphae) in Thermanox or polystyrene coverslips as seen in reports by Chew et al. (26). Interestingly, *in vivo* study reports by Zahran et al. (27) in intrauterine devices (IUD) and Kucharíková et al. (25) in a rat subcutaneous model also have shown pseudohyphal growth. A study report by Sasani et al. (28) showed that C. glabrata forms pseudohyphae in biofilms *in vivo* upon exposure to CO_2_, an *in vivo* factor (25, 27, 28). Our method of culturing can closely mimic the correct biofilm-forming environment for C. glabrata, which has shown dense yeast cells, EPS, and even pseudohyphae in low abundance. Our method also has accurately replicated the dense hyphae and EPS in the case of C. albicans. Apparently, our method of forming biofilm is addendum to the current state-of-the-art *in vitro* technique for thorough understanding of biofilm architecture of *Candida* species. This method can be exploited to understand the role of various signaling pathways and genes associated with the progression of biofilm, drug tolerance, and pathogenesis.

In the era of increased drug resistance and drug repurposing, robust drug screening methods are required. Further, our method of culturing with other methods like XTT in 96-well plates can solve the purpose of biochip to determine the effectivity of many drugs in combination or alone against *in vitro* biofilm. Nevertheless, this novel method mimicking *in vivo* can be used to dissect the heterogenicity of aggressive biofilm formation by C. auris. Thus, this methodology can be a highly potent drug screening approach for the rapid identification of promising novel antifungals.

## MATERIALS AND METHODS

### Strains and growth conditions.

Stocks of C. auris (CBS10913T), C. albicans (SC5314), and C. glabrata (BG2) were kept at −80°C. These strains were revived at 30°C overnight in yeast extract-peptone-dextrose (YPD).

### Coverslip coating.

**(i) Gelatin preparation.** One gram of bovine skin-derived gelatin was dissolved in 25 mL of ice-cold distilled water. The solution was vortexed to homogenize the mixture. The colloid was centrifuged at 1,000 × *g* for 10 min at 4°C. Subsequently, vortexing and centrifugation were done ~10 times or until the supernatant was clear. The washed gelatin was added to 100 mL of tissue culture grade water, making it ~1% wt/vol in a glass flask followed by autoclaving (Fig. 1A). This was stored at −20°C for further use.

**(ii) Coverslip treatment.** The 14-mm round coverslips were taken for electron microscopy, and 4 packs of coverslips were autoclaved for further use. The coverslips were treated with 0.3 N HCl and left for 20 to 24 h with frequent swirling in between. The acid was discarded and washed 4 times with 50 mL autoclaved water. Further coverslips were treated with 100% ethanol for 30 min. Coverslips were dried in a hot air oven. Two percent (3-aminopropyl)triethoxysilane was prepared in acetone, and coverslips were added to the solution one by one and incubated at room temperature for 5 min. The solution was discarded and washed with 50 mL autoclaved water ~3 to 4 times. The coverslips were dried in the hot-air oven again.

The chemically treated coverslips were taken out with sterile forceps and dipped in the relatively hot gelatin gel to ensure the uniform distribution of gelatin over the coverslip. Excess gelatin was soaked in low-lint tissues and left to dry. The process of gelatin coating, drying, and glutaraldehyde coating was repeated with each coverslip. After drying, finally, the coverslips were dipped in water and then soaked in low-lint tissue paper, stored between paper stacks, and kept at −20°C for further use (Fig. 1B).

### Biofilm formation.

Biofilms were formed on the gelatin-coated coverslips. For C. auris and C. glabrata RPMI 1640 was used, and for C. albicans RPMI 1640 with MOPS was used. In a 6-well plate, 3 mL medium (or in a 24-well plate, 1 mL medium) in each well was filled, and the coverslips were placed such that the coverslips stay at the bottom of the well and biofilm grows on top of the coverslips. From overnight grown primary cultures, secondary cultures were inoculated at an OD_600_ of 0.1. The cells were harvested after they reached an OD_600_ of ≤0.8. The cells were pelleted and resuspended in PBS. An OD_600_ of 0.5 was set in 3 mL medium poured inside a 6-well plate and incubated for 2.5 h at 37°C for adhering cells to the coverslips. C. auris treatment groups were treated with 14 μg/mL fluconazole at the 6th hour and 12th hour of postadherence incubation. For C. albicans and C. glabrata, no treatments were given. All of the biofilms were incubated for a total of 48 h.

### Calcofluor-white staining of biofilms.

After 48 h, the biofilm-containing coverslips were washed with 1× PBS buffer. The biofilm has formed on the top of the coverslips. Calcofluor-white stain (18909; Sigma-Aldrich) drop was put on the slide. The coverslip was placed such that the side on which the biofilm has formed faces the stain. The coverslip was sealed with transparent nail paint before visualization in the confocal microscope.

### LIVE/DEAD staining of biofilms.

After 48 h, the biofilm-containing coverslips were washed with 1× sodium-HEPES supplemented with 2% glucose buffer. A LIVE/DEAD *Funga*light yeast viability kit from Thermo Fisher scientific was used. After washing, 2 mL of sodium-HEPES supplemented with 2% glucose buffer was poured into each well. The stains were added at a concentration of 1 μL/mL. Coverslips were immersed in the solution and incubated at 37°C for at least 30 min. After incubation, the staining solution was discarded and washed with sodium-HEPES supplemented with 2% glucose buffer. The wet coverslip was placed such that the biofilm side faces the slide. The coverslips were sealed using transparent nail paint before visualization in Nikon Ti2.

### Imaging and analysis.

Imaging was carried out using a Leica SP5 confocal laser scanning microscope at 20× objective and using a 0.6-μm step size. In a confocal microscope, z-stacks of the biofilms were taken at a 0.6-μm stack width, and the stacks were taken from the top dark layer to the bottom dark layer, which is beyond the CFW signal. The 3D reconstruction of the stacks was done in FIJI. Further, the live and dead imaging was done using a Nikon Ti2 microscope at 40×. Analysis was done using the Las X suite and FIJI. The values were plotted in GraphPad Prism 9 for statistical analysis and graph plotting. The file was opened in FIJI 1.53V. Under the image option, brightness and contrast were set up to make the background dark, and under the plugin option, 3D viewer was chosen for 3D reconstruction.

### Preparation of sample for scanning electron microscopy.

The sample for scanning electron microscopy was processed according to a previous protocol with some modifications (29). After 48 h of biofilm formation on the gelatin-coated coverslip, the well containing the coverslip was given a wash with 0.15 M sodium cacodylate 2 times. A buffer was made by dissolving 2% paraformaldehyde, 2% glutaraldehyde, 0.15 M sodium cacodylate, and 0.15% alcian blue and was added to wells. This 6-well plate was incubated at 4°C for 16 to 22 h. Following the incubation, the coverslip was transferred to another 6-well plate and washed 2 times with 0.15 M sodium cacodylate. Subsequently, the coverslip was exposed to increasing concentrations of ethanol, starting with 25% for 5 min followed by 50% for 10 min, 70% for 15 min, 80% for 15 min, 95% for 30 min, and at last with absolute ethanol for 1 h. Then, the coverslips were vacuum dried for 3 h and subjected to absolute acetone for 4 h. The coverslips were further dried at 37°C for a whole day. The coverslips were then mounted on adhesive carbon tape. The fixed coverslips were coated with gold particles using an argon ion beam coater.

### XTT assay.

Biofilm was grown on gelatin-coated coverslips in a 24-well plate. Three groups were created as follows: the 6th hour treatment group, the 12th hour treatment group, and the untreated group. The 6th hour treatment group was treated with 14 μg/mL fluconazole at the 6th hour of incubation, and the 12th hour treatment group received the treatment at the 12th hour of incubation. The biofilm containing coverslips was transferred to a fresh 24-well plate. Five hundred microliters of 1 mg/mL XTT reagent (EZcount XTT cell assay kit from HiMedia Labs) was added. The plate was incubated for 3 h. Dimethyl sulfoxide (DMSO) was added to dissolve the formazan crystals that formed and diluted further to be detected by spectrophotometer. The absorbance was taken at 450 nm.

### Extraction of fatty acids.

Lipids were extracted using the established protocol with few modifications (30). Fatty acids were extracted from FBS using a mixture of MeOH and CHCl_3_ in the ratio of 1:2. This mixture was used with FBS in the ratio of 1:1 and vortexed for 5 min followed by a resting time of 1 min. This cycle was performed 4 times until the mixture was a complete emulsion. Subsequently, the emulsion was centrifuged to separate the layers. The top layer consisted of methanol and the bottom layer of chloroform. The lipids accumulated in the chloroform layer. The lipids were concentrated by volatilizing the chloroform using a vacuum concentrator, and the dry weight was measured. The lipids were then hydrolyzed using 1 M HCl at 50°C for 5 h with intermittent inversion and mixing.

### Preparation of sweat medium.

Sweat medium was prepared by modifying the SCIN sweat medium mentioned by Callewaert et al. (31). The modification was made by adjusting the concentration of fatty acid, glucose, sodium, magnesium, calcium, potassium, bicarbonate, and chloride mentioned previously (19, 32–35). The components of the modified sweat medium are mentioned in Table 1. These components were mixed in a magnetic stirrer and heated up to 60°C to mix the components and then filter sterilized using a 0.22-μm filter and kept at 4°C for further use.

### Data availability.

All data generated or analyzed during this current study are included in this article and its supporting information.
